# Validation of the Recovery Experience Questionnaire in a Lithuanian Healthcare Personnel

**DOI:** 10.3390/ijerph20032734

**Published:** 2023-02-03

**Authors:** Evaldas Kazlauskas, Austeja Dumarkaite, Odeta Gelezelyte, Auguste Nomeikaite, Paulina Zelviene

**Affiliations:** Center for Psychotraumatology, Institute of Psychology, Vilnius University, LT-01513 Vilnius, Lithuania

**Keywords:** healthcare, stress recovery, occupational stress, mental health, well-being

## Abstract

Healthcare workers (HCWs) often experience high levels of stress, anxiety, and depression due to high workloads and responsibilities in their professional activities. Therefore, recovery from work-related stress is highly important in HCWs. The Recovery Experience Questionnaire (REQ) is a 16-item self-reported measure covering four stress recovery domains: psychological detachment from work, relaxation, mastery, and control. The current study aimed to test the REQ’s psychometric properties in a sample of Lithuanian HCWs. In total, 471 HCWs from various healthcare institutions participated in this study. Confirmatory factor analysis (CFA) was used to test the structure of the REQ. We also used the Brief Patient Health Questionnaire (PHQ-4) and the World Health Organization Psychological Well-Being Index (WHO-5) to assess the mental health of the study participants. The CFA analysis supported the correlated four-factor structure of the REQ. Furthermore, we found significant correlations between the levels of REQ and anxiety, depression, and well-being. We conclude that the REQ is a valid measure that could be a useful tool in research on HCWs’ mental health. It could also be used in healthcare settings for the evaluation of well-being among healthcare staff.

## 1. Introduction

Healthcare workers (HCWs) are routinely exposed to high levels of occupational stress, often leading to burnout, anxiety, and depression. In particular, the COVID-19 (severe acute respiratory syndrome coronavirus 2, SARS-CoV-2) pandemic raised awareness of the highly demanding nature of the work of staff in healthcare systems [1,2]. Stressors of medical professions include long working hours, high levels of responsibility, less time for leisure activities compared with other professions [3], and moral injuries [4], among others.

In the context of occupational stress, it is important not only to understand the risk factors for mental disorders among medical professionals, but also to identify factors contributing to coping and recovery. Job stress recovery is a broad concept that includes the ability to relax, detach from work, and experience mastery and control [5,6,7]. Psychological detachment refers to the ability to distance oneself from work after working hours. Relaxation is the ability to let go of tension and return to a state of calm. Mastery is associated with the ability to achieve success in a hobby or non-work-related activity. Lastly, control refers to experiences of control over time and activities management in non-work hours [6,7]. The Recovery Experiences Questionnaire developed by Sonnentag and Fritz in 2007 is widely used for recovery experience research [5] and has been translated into multiple languages [7]. The four-factor structure and good psychometric properties of the Recovery Experience Questionnaire have been reported in different cultures and languages, e.g., Japan [8], Nepal [9], and Sweden [10]; however, it has not yet been validated in Lithuania.

Empirical studies reveal that individuals who score high on recovery experiences report better well-being [7]. It is known that recovery can mitigate the negative outcomes of job-related stress on mental health [11]. Stress recovery is an important set of skills that can improve occupational mental health [6,11], as well as reduce anxiety and depression. Initially proposed as a concept to study recovery in various occupational groups, it recently attracted interest in healthcare personnel studies [2,3], as recovery experiences are known to be associated with high job-related stress [7].

The aim of this study was to analyze the psychometric properties of the Recovery Experience Questionnaire (REQ) in help-seeking Lithuanian healthcare workers. First, based on previous studies [5,8,10], we tested the structural validity of the four-factor structure of the REQ with the four correlated latent factors of psychological detachment, relaxation, mastery, and control using confirmatory factor analysis (CFA). Second, we explored links between the REQ and anxiety, depression, as well as psychological well-being in the sample.

## 2. Materials and Methods

### 2.1. Participants and Procedure

The data for this study were extracted from two trials that tested the efficacy of internet stress recovery intervention for healthcare staff. More details on the study design have been reported in previous papers [12,13]. Only pre-test data collected prior to the intervention were used in this study.

In brief, healthcare professionals were invited to participate in an online psychological intervention program via social networks and emails to healthcare institutions across Lithuania. Participants from various healthcare institutions interested in participating in the study logged into the secure online platform designed for empirical data collection and could enter the study after providing informed consent for participation online. Participants were recruited between April 2021 and April 2022.

In total, data from 471 healthcare workers were included in the current study. The sociodemographic characteristics of the sample are presented in Table 1. The mean age of the sample was 42.16 years, and the majority were women (96.2%). Around half of the sample (54.8%) had a higher workload than one full-time equivalent (FTE), and 60.3% had more than 10 years of work experience. The majority of the sample were nursing staff, with around one-fifth being medical doctors (see Table 1).

### 2.2. Measures

#### 2.2.1. The Recovery Experiences Questionnaire

The Recovery Experiences Questionnaire (REQ) [5] is a 16-item self-reported scale designed to evaluate an individual’s skills in stress recovery in four domains: psychological detachment (4 items), relaxation (4 items), mastery (4 items), and control (4 items). The psychological detachment subscale measures how individuals are able to distance themselves from or forget work outside professional settings, with REQ items such as “I distance myself from work” or “I get a break from the demand at work”. The relaxation subscale measures how an individual is able to relax and includes items such as “I do relaxing things” or “I take time for leisure”. The mastery subscale measures the openness of an individual to new and challenging activities outside of work, with items such as “I do things that challenge me” or “I do something to broaden my horizons”. The fourth REQ subscale measures individuals’ perceptions of how much they can control their own lives and schedules, e.g., “I determine for myself how I will spend my time” or “I feel I can decide for myself what to do”.

Participants were asked to rate, on a five-point Likert scale, if they 0 = *Totally disagree* to 5 = *Totally agree* with each of the REQ items. The REQ scoring includes four subscales, which are the sums of the responses to the items comprising the subscale, and can range from 4 to 20, with higher scores indicating higher stress recovery experience. The sum of responses to all of the REQ items was used to compute the total score.

The back-translation procedure following the recommendations for the translation of psychological measures [14] was used to translate the REQ into the Lithuanian language using these steps: (1) two independent researchers, experts in the stress-related psychology research field (native Lithuanian and fluent English speakers), translated the REQ from English to Lithuanian; (2) two translators merged their individual translations of the REQ by discussing discrepancies in the two translations to provide the first version of the Lithuanian REQ; (3) back-translation to English was completed by an independent researcher fluent in English and Lithuanian, not associated with the research team, and not familiar with the REQ; (4) the REQ back-translation was checked by the authors of the measure; (5) the final REQ Lithuanian version was developed in communication with the authors of the REQ.

#### 2.2.2. The Patient Health Questionnaire-4

The brief four-item Patient Health Questionnaire (PHQ-4) [15] was used to screen for anxiety and depression in the sample. The PHQ-4 comprises two items measuring depression and two items measuring anxiety, and has been widely used for screening for depression and anxiety disorders in various populations. Participants were asked to indicate how often they had been bothered by the listed symptoms over the last two weeks on a four-point Likert scale ranging from 0 = *Not at all* to 3 = *Almost every day*. The scoring for anxiety is the sum of the responses to both anxiety items, and the depression score is the sum of the two depression items. The sum of the responses to all four items results in a total score of psychological distress, with a higher score indicating higher distress. Cut-off scores of ≥3 for anxiety and depression scores were used to assess probable anxiety or depression disorder. The internal consistency of the PHQ-4 was high in our study, with Cronbach’s alpha = 0.87.

#### 2.2.3. World Health Organization Well-Being Index-5

The five-item self-reported World Health Organization Well-being Index (WHO-5) was used to assess psychological well-being [16]. The WHO-5 has been translated into multiple languages and used widely in mental health research. Participants were asked to evaluate how they felt over the last two weeks and rate each item on a five-point Likert scale ranging from 0 = *At no time* to 5 = *All the time*. The total score of the WHO-5 is a sum of responses to all five items multiplied by four and can range from 0 to 100, with higher scores indicating higher well-being. The internal consistency of the WHO-5 in our study sample was high, with Cronbach’s alpha = 0.88.

### 2.3. Data Analysis

Confirmatory factor analysis (CFA) was used to test the structure of the Recovery Experiences Questionnaire (REQ). CFA was chosen as the recommended analytical tool [17] for testing the psychometric properties of measurement instruments. We tested a factor model with four correlated latent factors: (1) psychological detachment, (2) relaxation, (3) mastery, and (4) control. Several model fit indices widely used in the estimation of structural equation modeling (SEM) were applied to estimate the CFA model’s fit: (1) the Comparative Fit Index (CFI) [18], the Tucker–Lewis Index (TLI) [19], and the Root-Mean-Square Error of Approximation (RMSEA [20]. Based on recommendations provided by Kline [21], CFI/TLI values higher than 0.90 indicated an acceptable fit, and values higher than 0.95 represented a good fit; RMSEA values equal to or below 0.08 indicated an acceptable fit. We used a robust Weighted-Least-Square with Mean and Variance Adjusted (WLSMV) estimator in the analysis. Additionally, the composite reliability of the REQ factors based on the estimated factor loadings of the tested model was calculated; values above 0.60 showed acceptable internal reliability [22]. The dataset included no missing data. The CFA analysis was conducted with the Mplus 8.8 [23]. IBM SPSS Statistics 28 was used for the remaining analyses.

## 3. Results

### 3.1. Mental Health of the Sample

The healthcare workers who were included in the study reported high levels of anxiety and depression. Based on the PHQ-4 cut-off score, probable depression was observed in 45.9% of the sample, and 53.9% of the sample was at risk for anxiety disorder.

### 3.2. Factor Structure of the REQ

Initial CFA analysis revealed that the correlated four-factor model did not fit the data well, with RMSEA = 0.136 (95% CI [0.128, 0.144]), CFI/TLI = 0.921/0.903, SRMR = 0.064, χ^2^(98) = 948.36, *p* < 0.001). Further, seven residual error covariances were added based on the high-modification indices suggested by Mplus. With these modifications, CFA analysis supported the structural validity of the REQ four-factor model, with RMSEA = 0.08 (95% CI [0.071, 0.088]), CFI/TLI = 0.975/0.968, SRMR = 0.039, and χ^2^(91) = 361.91, *p* < 0.001. Overall, the model fit indices indicated a good model fit, with RMSEA showing an acceptable fit and CFI/TLI demonstrating a good model fit.

**Figure 1:**
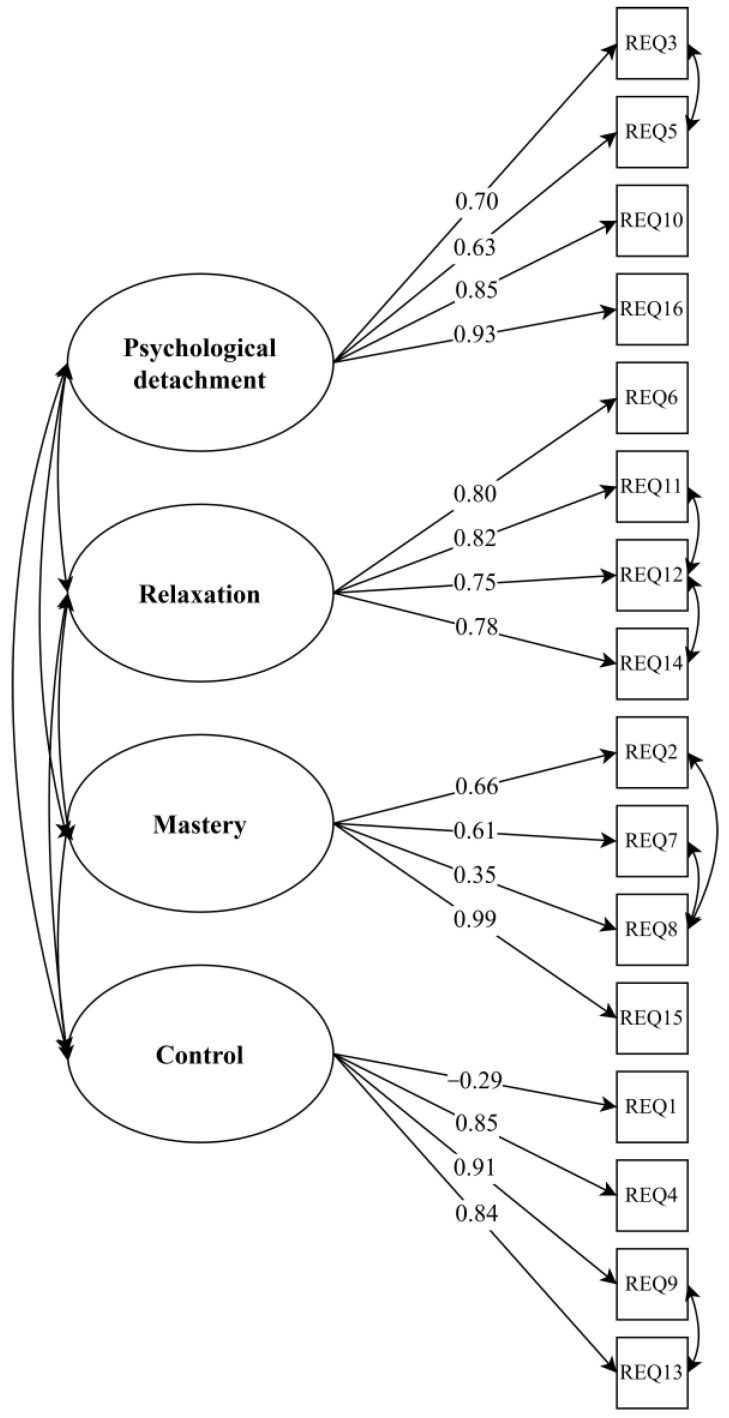
Confirmatory factor analysis model of the Recovery Experiences Questionnaire.

The correlations between latent factors were all significant at *p* < 0.001 and ranged between 0.45 and 0.77, namely, between psychological detachment and relaxation (0.77), mastery (0.45), and control (0.49); between relaxation and mastery (0.68) and control (0.76); and between mastery and control (0.52). All factor loadings of the items are presented in Figure 1. The factor loadings were all significant at *p* < 0.001 and ranged from 0.29 to 0.99. Overall, all item factor loadings were high, except for REQ item 8 “I do things that challenge me” on the mastery latent factor (0.35) and item 1 “I feel like I can decide for myself what to do” (0.29) on the control latent factor. The means and correlations between the REQ item scores are presented in Supplementary Table S1.

Following the CFA analysis, which supported the structure of the REQ measure, we assessed the composite reliability of the REQ factors. The estimates of composite reliability derived from the model estimates indicated acceptable levels of internal reliability for all four factors: psychological detachment (0.82), relaxation (0.87), mastery (0.77), and control (0.91).

### 3.3. Correlations between the REQ and Mental Health Indicators

To further estimate the validity of the REQ, we computed bivariate correlations between the stress recovery experiences and mental health measures included in the study. Descriptive statistics and bivariate correlations between all of the measures are presented in Table 2. We found significant correlations between the REQ and all other mental health measures. Overall, the total REQ and all REQ domains were positively correlated with psychological well-being, with correlations ranging from 0.40 to 0.57. Furthermore, the subscales and the total score of the REQ were significantly negatively correlated with anxiety, depression, and general distress levels, with correlations ranging from −0.25 to −0.46.

### 3.4. Recovery Experiences in Different HCWs Groups

Recovery experience among medical doctors was significantly lower compared with nurses and other HCW professional groups in all stress recovery domains. The following are the scores for medical doctors and other professionals, respectively: psychological detachment, *M* = 9.98 (*SD* = 2.93) and *M* = 10.93 (*SD* = 3.45) *t*(469) = 2.48, *p* = 0.013; relaxation, *M* = 12.47 (*SD* = 2.81) and *M* = 13.33 (*SD* = 3.15) *t*(469) = 2.42, *p* = 0.016; mastery *M* = 11.79 (*SD* = 3.58) and *M* = 12.85 (*SD* = 3.13) *t*(469) = 2.88, *p* = 0.004, control *M* = 13.59 (*SD* = 2.72) and *M* = 13.59 (*SD* = 2.72) *t*(469) = 2.84, *p* = 0.005; and the total REQ *M* = 47.83 (*SD* = 8.58) and *M* = 51.62 (*SD* = 9.95) *t*(469) = 3.40, *p* < 0.001. We did not find any effects of workload in terms of part-time, full-time, or exceeding full-time work on recovery experiences in our sample.

### 3.5. Predictors of Recovery Experiences

Additionally, we conducted multiple linear regression analysis to estimate how recovery experiences predicted psychological well-being, controlling for age, gender, workload, and doctors vs. other professions (*F*(8, 416) = 28.28, *p* < 0.001, *R*^2^ = 35.2%). All recovery experience domains were significant predictors of psychological well-being: psychological detachment (β = 0.14, *p* = 0.004), relaxation (β = 0.22, *p* < 0.001), mastery (β = 0.20, *p* < 0.001), and control (β = 0.16, *p* = 0.002).

## 4. Discussion

This study provided promising findings on the validity of the Lithuanian version of the Recovery Experiences Questionnaire among healthcare workers. We found support for the factorial validity and high internal consistency, similar to the findings of the previous REQ validation studies in other languages [8,10]. Moreover, we found that the REQ scores were highly positively correlated with psychological well-being and negatively correlated with psychological distress. The correlated four-factor model, which we found to have a good fit, was in line with previous studies, e.g., [3,4,5].

As the concept of recovery experiences is relatively new in HCWs research [2], our study raises several questions and potential areas for future studies. It has been hypothesized that job stress recovery skills could be one of the underlying mechanisms explaining how burnout or other occupation-related psychological difficulties develop [3,6]. If there is a lack of stress recovery skills, especially in detachment from the work domain, there might be a higher risk for mental disorders in response to a high workload. We could assume that stress recovery is closely related to other internal resources, such as coping with stress or resilience. Therefore, future studies could look into the links between stress recovery experiences and coping or resilience among HCW samples.

The need to study the mental health of HCWs has been demonstrated by the results of our study, which are very much in line with those of previous research reporting high levels of distress and mental disorders in healthcare staff [1,2,24]. Our study revealed intense psychological distress in HCWs, with almost half of the study sample reporting high levels of depression and anxiety. The majority of the sample were highly experienced professionals with more than 10 years of job experience. However, it is important to note that our sample was help-seeking and self-referred to psychological intervention, so, naturally, psychological difficulties in such a sample might be elevated. Nevertheless, the current study provides information indicating that research on the mental health of medical personnel is highly important.

One of the most interesting and relevant findings of our study was that doctors had reported lower stress recovery compared with other healthcare professionals (including nurses, assistant nurses, and public health specialists). We hypothesize that doctors may face more responsibilities and pressures in decision-making, which may interfere greatly with their efficient recovery from stress. Additionally, the timing of data collection could be of great importance, as data were collected during the COVID-19 pandemic, and doctors faced an enormous additional workload amid the pandemic. However, in other studies, opposite results were found, indicating that medical doctors may have higher levels of resilience as compared with nurses [25] or more adaptive coping strategies [26], or that doctors and nurses use coping strategies similarly [27]. Thus, future studies should aim to explore stress recovery in medical doctors. Several limitations of this study should be outlined. First, the sample was help-seeking, with higher levels of psychological distress, in contrast to previous studies of HCWs in Lithuania [24]. Moreover, the participants were predominantly female, which could have had an impact on the study’s findings. Additionally, the study was cross-sectional in design. Therefore, we could not identify the trajectories of recovery experiences and dynamic associations with mental health indicators over time. Previous studies indicated that psychosocial interventions could increase stress recovery experiences [12,28,29], revealing that they can change over time. More longitudinal studies are needed to evaluate changes in recovery experiences.

In addition to this, this study was conducted during the COVID-19 outbreak, when HCWs had even more demanding and challenging work conditions due to the lockdowns, the high number of COVID-19 infections, and uncertainty related to the ongoing pandemic. However, the specific aim of this study was to test the psychometric properties of the REQ. Even though the sampling and specific context could have had an impact on the study’s findings, we found strong support for the validity and reliability of the Lithuanian version of the REQ. The main implication of this study is the availability of an internationally recognized measure that could foster stress recovery research in Lithuania and enable a comparison of the findings with studies conducted in other countries. Future studies should test the properties of the REQ on other samples, providing even more information on the psychometric characteristics of the measure.

## 5. Conclusions

Our study provided further support for the validity of the REQ and that it could be a useful measure in HCWs’ mental health research. The current study indicates that recovery from stress is associated with psychological well-being and distress in HCWs, and that the REQ can be used in healthcare settings for the evaluation of well-being and the exploration of underlying mechanisms of burnout and distress. We conclude that the REQ is a valid measure for recovery experience and can be used in the Lithuanian context.

## Figures and Tables

**Table 1:** Sociodemographic characteristics of the sample (*N* = 471).

	*N*	%
Gender		
Female	453	96.2
Male	18	3.8
Age		
Mean (*SD*)	42.16 (12.03)	
Range	20–70	
Education		
Secondary or lower	10	2.1
Professional college	186	39.5
University degree	275	58.4
Profession		
Doctor	95	20.2
Nurse	294	62.4
Assistant nurse	12	2.5
Public health specialist	11	2.3
Other	59	12.5
Workload		
Part-time	28	5.9
Full-time	185	39.3
Exceeding full-time	258	54.8
Work experience		
<2 years	67	14.2
2–5 years	57	12.1
6–10 years	63	13.4
>10 years	284	60.3

**Table 2:** Descriptive statistics and bivariate correlations between the study measures (*N* = 471).

Measure	*M* (*SD*)	1	1.1	1.2	1.3	1.4	2	2.1	2.2
1. Recovery experiences	50.86 (9.80)	-							
1.1. Psychological detachment	10.74 (3.37)	0.76	-						
1.2. Relaxation	13.16 (3.10)	0.86	0.58	-					
1.3. Mastery	12.64 (3.25)	0.69	0.32	0.43	-				
1.4. Control	14.31 (3.02)	0.78	0.40	0.65	0.38	-			
2. Distress	5.62 (3.01)	−0.46	−0.40	−0.42	−0.30	−0.30	-		
2.1. Anxiety	2.91 (1.71)	−0.40	−0.37	−0.37	−0.25	−0.25	0.93	-	
2.2. Depression	2.71 (1.56)	−0.45	−0.36	−0.41	−0.30	−0.31	0.91	0.68	-
3. Psychological well-being	37.20 (19.03)	0.57	0.40	0.50	0.42	0.45	−0.63	−0.56	−0.61

All correlations are significant at *p* < 0.01.

## Data Availability

The dataset used in the data analysis supporting our study findings can be obtained from the corresponding author upon reasonable request.

## Supplementary Materials

The following supporting information can be downloaded at: https://www.mdpi.com/article/10.3390/ijerph20032734/s1, Table S1: Descriptive statistics and inter-correlations of the Recovery Experience Questionnaire items.
