# Supplementary material for: Validation of the Recovery Experience Questionnaire in a Lithuanian Healthcare Personnel
Source: Int J Environ Res Public Health. 2023 Feb 3;20(3):2734. doi: 10.3390/ijerph20032734 (PMC9915394; doi:10.3390/ijerph20032734)
Supplement: Supplementary file 1 [file ijerph-20-02734-s001.zip › ijerph-2197430-supplementary.pdf]

SUPPLEMENTARY MATERIAL

Table S1. Descriptive statistics and inter-correlations of the Recovery Experience Questionnaire items

No	Items	M (*SD*)	1	2	3	4	5	6	7	8	9	10	11	12	13	14	15
1	Item 1	3.51 (1.02)	-														
2	Item 2	3.26 (1.09)	.28**	-													
3	Item 3	2.51 (1.06)	.20**	.22**	-												
4	Item 4	3.48 (0.94)	.63**	.24**	.30**	-											
5	Item 5	2.20 (0.97)	.16**	.15**	.66**	.21**	-										
6	Item 6	2.89 (0.98)	.45**	.28**	.43**	.44**	.40**	-									
7	Item 7	2.99 (1.01)	.19**	.47**	.24**	.17**	.17**	.27**	-								
8	Item 8	2.96 (1.04)	.10*	.51**	.26**	.09*	.18**	.13**	.60**	-							
9	Item 9	3.54 (0.95)	.64**	.29**	.26**	.70**	.21**	.46**	.27**	.15**	-						
10	Item 10	2.99 (0.99)	.26**	.18**	.55**	.31**	.52**	.55**	.17**	.08	.36**	-					
11	Item 11	3.44 (0.90)	.47**	.33**	.35**	.46**	.26**	.53**	.35**	.15**	.52**	.45**	-				
12	Item 12	3.34 (0.94)	.41**	.26**	.28**	.39**	.23**	.53**	.27**	.12**	.44**	.42**	.76**	-			
13	Item 13	3.79 (0.78)	.34**	.28**	.22**	.44**	.22**	.36**	.23**	.11*	.46**	.29**	.44**	.41**	-		
14	Item 14	3.49 (0.87)	.42**	.23**	.26**	.39**	.22**	.52**	.21**	.02	.47**	.46**	.60**	.62**	.41**	-	
15	Item 15	3.43 (0.94)	.36**	.54**	.25**	.32**	.17**	.41**	.47**	.37**	.42**	.27**	.50**	.50**	.37**	.52**	-
16	Item 16	3.05 (1.00)	.27**	.20**	.59**	.35**	.55**	.55**	.24**	.14**	.37**	.68**	.49**	.48**	.36**	.50**	.40**
